# Correlation Between Serum Uric Acid and Renal Function in Patients With Stable Coronary Artery Disease and Type 2 Diabetes

**DOI:** 10.14740/jocmr1909w

**Published:** 2014-09-09

**Authors:** Zhong Chen, Zhen Ding, Cong Fu, Chaojun Yu, Genshan Ma

**Affiliations:** a Department of Cardiology, The Affiliated Sixth People’s Hospital of Shanghai Jiao Tong University, No. 600 Yishan Road, Shanghai 200233, China; b Department of Cardiology, Zhenjiang First People’s Hospital, Zhenjiang 212002, China; c Department of Cardiology, The Affiliated Zhongda Hospital of Southeast University, No. 87 Dingjiaqiao, Nanjing 210009, China

**Keywords:** Stable coronary artery disease, Estimated glomerular filtration rate, Type 2 diabetes mellitus, Uric acid, Follow-up

## Abstract

**Background:**

The aim of this study was to investigate the relationship between serum uric acid and renal function, expressed as estimated glomerular filtration rate (eGFR), in patients with stable coronary artery disease (CAD) and type 2 diabetes mellitus (T2DM) in China.

**Methods:**

Serum levels of uric acid and creatinine were determined in 526 enrolled patients diagnosed with stable CAD and T2DM. To assess renal function, eGFR was calculated using a modified MDRD formula suitable for the Chinese population. Patients’ anthropometric measurements were collected using standardized protocols, and 6-month follow-up results were collected and analyzed. Pearson’s correlation coefficient r was calculated and multivariate analysis was performed to evaluate the relationship between uric acid and renal function. Patients with eGFR < 60 mL/min/1.73 m^2^ were classified as having reduced renal function or chronic kidney disease (CKD) in this study.

**Results:**

Uric acid levels were negatively associated with eGFR (P = 0.002), especially in patients with CKD (eGFR < 60 mL/min/1.73 m^2^) (P < 0.001). In patients with reduced renal function, the risk in the highest quartile of uric acid levels was higher than in the lowest quartile (odds ratio 9.18, 95% confidence interval: 4.01 - 21.01, P < 0.001). These associations remained after multiple adjustments for potential confounders and were recapitulated after 6-month follow-up.

**Conclusions:**

Serum uric acid level is negatively associated with renal function, as assessed by eGFR, and serves as an independent predictor for CKD in patients with stable CAD and T2DM.

## Introduction

Chronic kidney disease (CKD) has become a public health problem worldwide, and is associated with increased risk of cardiovascular morbidity and mortality [1-4]. For clinical and epidemiological study purposes, CKD has been classified based on estimated glomerular filtration rate (eGFR) [5]. Reduced GFR, characterized by GFR < 60 mL/min/1.73 m^2^, has been associated with poorer cardiovascular and renal prognosis in a variety of populations [1]. Based on these studies, decreasing eGFR value has potential as a readily obtainable and inexpensive novel biomarker for evaluating the progression of atherothrombotic disease [6].

Serum levels of uric acid, the end product of human purine metabolism, have attracted renewed attention recently based on their association with cardiovascular and renal disease, hypertension [7], metabolic syndrome [8] and carotid atherosclerosis [8, 9]. Moreover, a number of large prospective investigations have demonstrated that serum uric acid is an independent risk factor for the progression of renal dysfunction [10-12] and cardiovascular events [13-15].

In China the prevalence of type 2 diabetes mellitus (T2DM) has increased so rapidly that nearly 10% of Chinese adults had T2DM in 2008, compared with 1% in 1980 [16, 17]. Patients with T2DM have an increased incidence of atherosclerotic cardiovascular disease, and individuals diagnosed with both coronary artery disease (CAD) and T2DM belong to a very-high-risk population that warrants increased attention [18-22]. There is little information on whether serum uric acid is an independent risk factor or a predictor of future renal function in this very-high-risk cohort in the Chinese population. Accordingly, the aim of this study was to investigate the relationship between baseline serum uric acid levels and baseline and follow-up renal function in patients with stable CAD and T2DM in a Chinese population.

## Materials and Methods

### Ethics statement

The present investigation was conducted according to the principles expressed in the Declaration of Helsinki. The study protocol was approved by the Medical Ethics Committee of the Affiliated Zhongda Hospital of Southeast University and written informed consent was obtained from all participants.

### Study population

This study was performed in a population of 526 patients diagnosed with stable CAD and T2DM who were hospitalized in the Affiliated Zhongda Hospital of Southeast University between November 2009 and December 2010. Patients underwent elective coronary angiography (CAG) according to the Judkins technique based on a report of chest discomfort. CAD was diagnosed by CAG with stenosis that obstructed at least one main coronary lumen by ≥ 50%, and those individuals with a documented prior history of myocardial infarction. Patients with unstable anginal pectoris, acute myocardial infarction, congenital heart disease, multiple aorto-arteritis, syndrome X, and severe liver or kidney disease, were excluded from the study.

### Laboratory measurements and data collection

Plasma concentrations of total cholesterol (TC), low-density lipoprotein cholesterol (LDL-C), high-density lipoprotein cholesterol (HDL-C), apolipoprotein A1 (apo A1) and apolipoprotein B (apo B), fasting blood sugar (FBS) and hemoglobin were determined using standard methods with a chemical analyzer (Beckman Coulter Synchron Clinical System LX20, Fullerton, CA, USA). Serum uric acid levels were measured by urate oxidase and serum creatinine was measured using a kinetic alkaline picrate assay. The eGFR was calculated using a modified MDRD formula suitable for the Chinese population [4]. Patients with eGFR < 60 mL/min/1.73 m^2^ were classified as having reduced renal function or “CKD” in this study. All subjects were divided into four groups based on their serum uric acid levels.

A questionnaire was completed based on the medical records of each participant. Data collected from each individual included hypertension status, drug usage and smoking status. Anthropometric measurements and blood pressure were determined according to standard protocols. Hypertension was defined as blood pressure ≥ 140/90 mm Hg and/or use of anti-hypertensive medications. Subjects with a history of T2DM, those receiving anti-diabetic medications, and those with confirmed FBS > 126 mg/dL (7.0 mmol/L), were categorized as having T2DM. Subjects who smoked at least one cigarette per day at the time of enrollment were considered as smokers.

All participants were followed up from the date of discharge until the end of the study period (June 30, 2011). Six-month data were collected from patients via clinic visit. Twenty-three patients did not undergo follow-up, corresponding to an overall 6-month follow-up rate of 95.63%. The major follow-up issues were determination of the serum uric acid and creatinine levels.

### Statistical analyses

Statistical analyses were conducted using the SPSS 15.0 software (SPSS, Inc., Chicago, IL). Continuous variables were expressed as mean ± SD and categorical variables were represented by frequency and percentage. One-way ANOVA or Chi-square test was used to analyze differences between groups. Serum uric acid levels were depicted according to the difference values of eGFR. Pearson’s correlation coefficient r was calculated to test for associations between uric acid and eGFR. Multivariate logistic regression models were used to estimate the odds ratios (ORs) for renal dysfunction. Potential confounding variables including age, gender, smoking, hypertension, LDL-C, apo A1 and apo B, and medicine such as insulin, anti-diabetic pills, diuretic, angiotension converting enzyme inhibitors (ACEI) and statins use were controlled in the regression models. Two-tailed P values < 0.05 were considered to be statistically significant.

## Results

### Baseline characteristics

The patient cohort consisted of 526 individuals diagnosed with both stable CAD patients and T2DM (mean age: 69.31 ± 9.91 years), 257 (48.85%) male and 269 (51.15%) female. Baseline demographic and medical characteristics for both genders, both combined and divided by uric acid quartiles, are listed in Table 1. Compared with patients in the lowest uric acid quartiles (Q1), patients in the highest uric acid quartiles (Q4) were more likely to be older and have higher levels of serum creatinine and lower values of eGFR, LDL-C, apo A1 and apo B (all P < 0.05 - 0.01). No significant differences were observed with respect to hypertension status, gender, smoking status, use of insulin, diabetic pills, diuretic, ACEI and statins and mean values of FBS, HbA1C, TC and hemoglobin, between the four groups (all P > 0.05).

**Table 1:** Baseline Characteristics of Diabetic Patients With Stable CAD According to Uric Acid Quartiles

Variables	Uric acid			
	Q1 (n = 132)	Q2 (n = 131)	Q3 (n = 132)	Q4 (n = 131)
Uric acid, µmol/L	189 (< 246)	278 (247 - 307)^†^	334 (308 - 365)^†^	468 (> 365)^†^
Age, years (mean ± SD)	67.42 ± 10.49	68.23 ± 8.97	69.45 ± 9.97	72.16 ± 9.61^†^
Male, n (%)	55 (41.70)	62 (47.30)	63 (47.70)	77 (58.80)
Smoking, n (%)	29 (22.00)	35 (26.70)	35 (26.50)	34 (26.00)
Hypertension, n (%)	96 (72.70)	109 (83.20)	105 (79.50)	114 (87.00)
HbA1C (%)	8.35 ± 3.41	8.36 ± 3.15	8.38 ± 3.28	8.37 ± 3.64
FBS, mmol/L	6.77 ± 4.10	6.75 ± 4.27	6.78 ± 3.87	6.81 ± 4.23
Hemoglobin, g/L	127.69 ± 23.86	127.36 ± 30.69	127.71 ± 31.79	122.69 ± 29.03
Serum creatinine, µmol/L	72.14 ± 35.16	80.78 ± 40.44	83.41 ± 30.21*	116.83 ± 62.05^†^
eGFR, mL/min/1.73 m^2^	52.27 ± 18.71	53.06 ± 16.10	52.39 ± 15.79	45.17 ± 16.76*
Insulin (%)	47 (35.60)	50 (38.20)	42 (31.80)	42 (32.10)
Anti-diabetic pills (%)	89 (67.40)	80 (61.10)	89 (67.40)	82 (62.60)
Diuretic (%)	13 (9.80)	22 (16.80)	20 (15.20)	46 (35.1)
ACEI (%)	68 (51.50)	75 (57.30)	79 (59.80)	71 (54.20)
Statins (%)	99 (75.00)	101 (77.10)	102 (77.30)	96 (73.30)
HDL-C, mmol/L	1.13 ± 0.42	1.00 ± 0.31*	1.04 ± 0.36	1.02 ± 0.33
LDL-C, mmol/L	2.74 ± 1.11	2.54 ± 0.97	2.85 ± 1.65	2.23 ± 0.94^†^
Total cholesterol, mmol/L	3.97 ± 1.54	4.01 ± 1.48	4.02 ± 1.51	4.01 ± 1.55
apo A1, g/L	1.04 ± 0.24	0.97 ± 0.29*	1.00 ± 0.32	0.90 ± 0.32^†^
apo B, g/L	0.79 ± 0.28	0.74 ± 0.27	0.80 ± 0.37	0.68 ± 0.26^†^
apo B/apo A1	0.77 ± 0.25	0.78 ± 0.24	0.81 ± 0.25	0.78 ± 0.22

P < 0.05, ^†^P < 0.001 compared with Q1. ACEI: angiotension converting enzyme inhibitor; Apo A1: apolipoprotein A1; Apo B: apolipoprotein B; CAD: coronary artery disease; eGFR: estimated glomerular filtration rate; FBS: fasting blood sugar; HDL-C: high-density lipoprotein cholesterol; LDL-C: low-density lipoprotein cholesterol.

### Association between serum uric acid levels and eGFR

**Figure 1:**
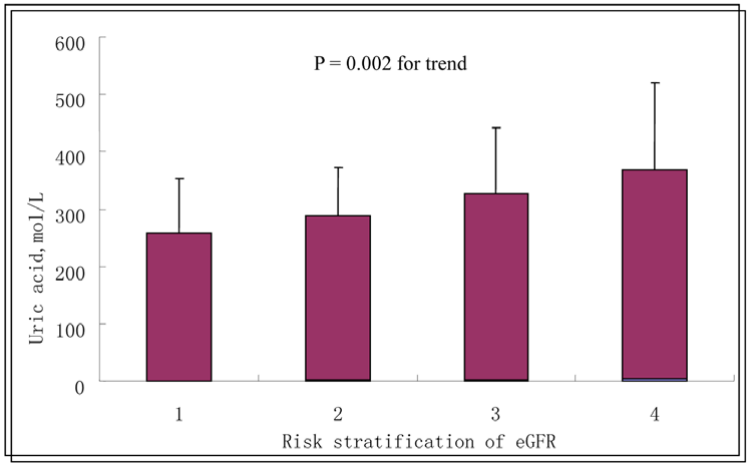
Serum uric acid levels according to the grade of eGFR. Data are shown as means ± SE with no adjustment; P = 0.002 for trend. 1: eGFR ≥ 90 mL/min/1.73 m^2^; 2: eGFR 60 - 89 mL/min/1.73 m^2^; 3: eGFR 30 - 59 mL/min/1.73 m^2^; 4: eGFR < 30 mL/min/1.73 m^2^.

When patients were divided into four groups according to eGFR values, namely, eGFR ≥ 90, 60 - 89, 30 - 59, and < 30 mL/min/1.73 m^2^. Unadjusted mean serum uric acid concentrations, in μmol/L (SE), were 256.30 (95.10) (eGFR ≥ 90); 285.70 (84.6) (eGFR = 60 - 89); 323.82 (114.19) (eGFR = 30 - 59); and 363.53 (153.08) (eGFR ≥ 90), indicating a positive correlation between serum uric acid levels and grade of eGFR (Fig. 1).

**Figure 2:**
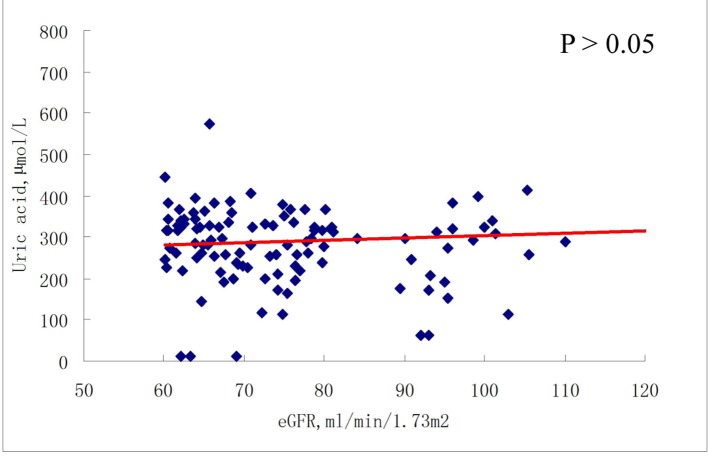
Correlations between baseline serum uric acid and eGFR (≥ 60 mL/min/1.73 m^2^).

**Figure 3:**
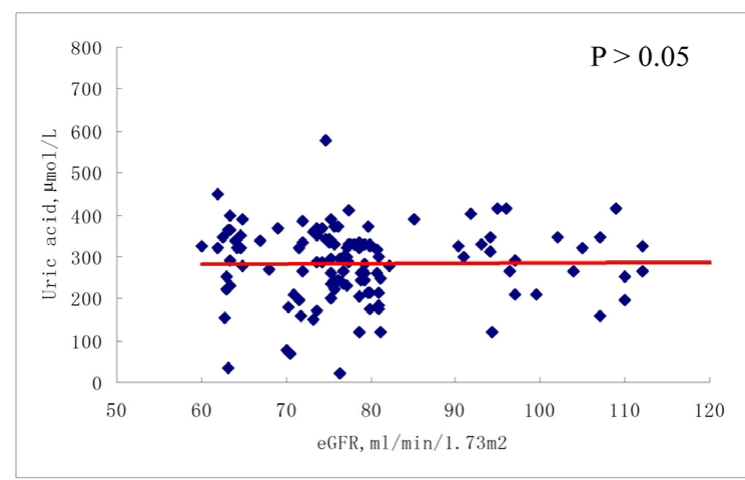
Correlations between serum uric acid and eGFR (≥ 60 mL/min/1.73 m^2^) after 6-month follow-up.

**Figure 4:**
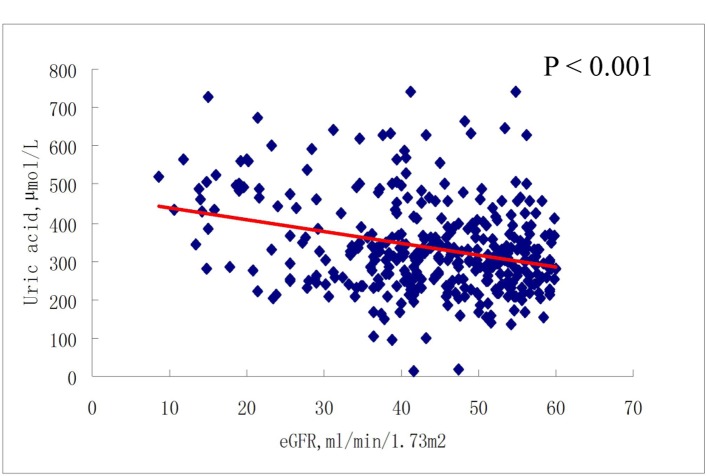
Correlations between baseline serum uric acid and eGFR (< 60 mL/min/1.73 m^2^).

**Figure 5:**
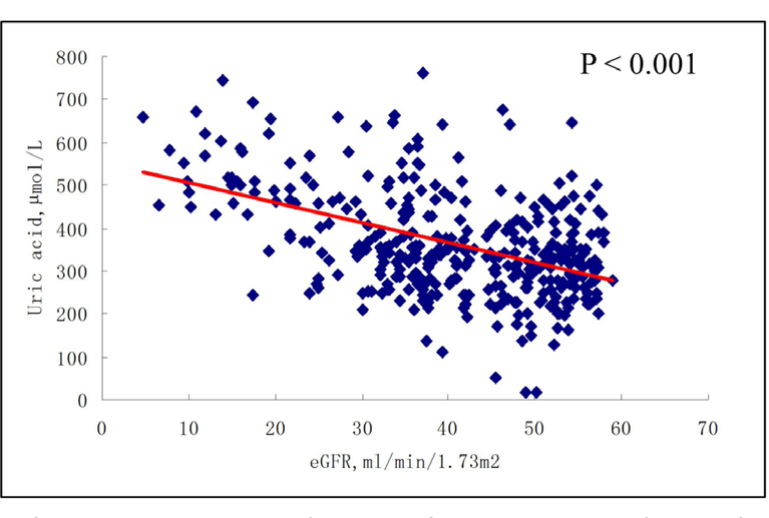
Correlations between plasma levels of uric acid and eGFR (< 60 mL/min/1.73 m^2^) after 6-month follow-up.

As shown in Figures 2 and 3, there was no correlation between serum uric acid and eGFR in participants with eGFR ≥ 60 mL/min/1.73 m^2^, either at baseline or at 6-month follow-up. In contrast, in participants with eGFR < 60 mL/min/1.73 m^2^, serum uric acid was negatively correlated with eGFR, at both baseline and 6-month follow-up (all P < 0.001) (Fig. 4, 5).

### Association between serum uric acid levels and CKD

In the highest uric acid quartile, the OR for the association between serum uric acid concentration and CKD with no adjustment was 9.18 (95% confidence interval (CI): 4.01 - 21.01) at baseline and 10.01 (95% CI: 4.54 - 22.07) after 6-month follow-up (Table 2). After adjusting for age, gender, smoking status, hypertension status, diuretic status, use of insulin or anti-diabetic pills, ACEI, statins, LDL-C, apo A1 and apo B, the OR was 7.01 (95% CI: 3.26 - 15.09) at baseline and 9.10 (95% CI: 3.93 - 21.09) after 6-month follow-up (Table 2).

**Table 2:** Adjusted ORs and 95% CIs for eGFR < 60 mL/min/1.73 m^2^ According to Uric Acid Quartiles

eGFR < 60 mL/min/1.73 m^2^	ORs (95% CI)			
	Q1	Q2	Q3	Q4
Model 1	1.0	0.96 (0.54 - 1.70)	2.23 (1.20 - 4.16)*	9.18 (4.01 - 21.01)^†^
Model 2	1.0	0.98 (0.56 - 1.71)	2.20 (1.19 - 4.07)*	8.98 (3.95 - 20.43)^†^
Model 3	1.0	0.85 (0.51 - 1.40)	2.01 (1.19 - 3.66)*	7.01 (3.26 - 15.09)^†^
Model 4 6 months later	1.0	1.23 (0.74 - 2.03)	2.93 (1.68 - 5.11)^†^	10.01 (4.54 - 22.07)^†^
Model 5 6 months later	1.0	1.31 (0.76 - 2.26)	2.52 (1.39 - 4.57)*	9.29 (4.08 - 21.17)^†^
Model 6 6 months later	1.0	1.27 (0.73 - 2.23)	2.75 (1.47 - 5.14)*	9.10 (3.93 - 21.09)^†^

*P < 0.05, ^†^P < 0.01. Model 1 no adjustment. Model 2 adjusted for age, gender, smoking, hypertension and diuretic. Model 3 further adjusted for insulin, diabetic pills, diuretic, ACEI, statins, LDL-C, apo A1 and apo B. Model 4 no adjustment. Model 5 adjusted for age, gender, smoking, hypertension and diuretic. Model 6 further adjusted for insulin, diabetic pills, diuretic, ACEI, statins, LDL-C, apo A1 and apo B. CI: confidence interval; ORs: odds ratios; other abbreviations see Table 1.

## Discussion

To our knowledge, this report is the first to study the association between serum uric acid and eGFR in individuals with both T2DM and stable CAD in the Chinese population. We found that serum uric acid is negatively associated with eGFR in the reduced renal function stage (< 60 mL/min/1.73 m^2^) and is an independent risk factor for CKD in individuals with T2DM and stable CAD. Moreover, patients with higher serum levels of uric acid are more likely to be older, and to have a higher value of serum creatinine. Furthermore, patients with higher serum levels of uric acid had higher ORs for CKD after 6-month follow-up.

It is well known that CAD is the leading cause of death among T2DM persons. Individuals diagnosed with both T2DM and CAD belong to a very-high risk group in terms of future cardiovascular risk, necessitating further risk stratification. In the present study, in order to analyze consistent, reliable data collected under “real-world” conditions, only individuals diagnosed with both T2DM and stable CAD at a single hospital were included. At baseline, compared with patients in the lowest uric acid quartiles, patients in the highest uric acid quartiles were more likely to be older, and to have higher serum levels of creatinine and lower eGFR values. Moreover, uric acid concentrations significantly increased from the first to the fourth grade of eGFR. This reflects to some extent the association between uric acid and renal function, and the fact that increasing age plays a role in the regression of renal function.

No significant differences were observed between the four groups based on serum uric acid levels with respect to hypertension, gender and smoking, statins usage and mean values of FBS, HbA1C, TC, hemoglobin and LVEF. Even so, we found a lower ratio of statin and ACEI use within the four groups (75.0-77.3% and 51.5-59.8% respectively) and the overall poor renal function might the main concern among physicians. This reflects the current status of the medical profession, in which physicians’ practice lags behind guidelines. We suggest that physicians should play a more active role in their patients’ medication and treatment, and that more should be done in our daily practice to improve the relationship between medication and the achievement of target goals.

Multiple logistic regression analysis of 7,078 individuals in the general population with normal eGFR (≥ 60 mL/min/1.73 m^2^) over an average 4.6-year follow-up period has shown that new-onset CKD is independently correlated with baseline uric acid levels after adjustment for co-existing factors [23]. Similar results were observed in the same study in subjects with normal uric acid levels [23]. In another study of 1,449 T2DM patients with normal kidney function and without overt proteinuria [10] , after a 5-year follow-up, and after adjusting for eGFR and potential confounders, hyperuricemia was found to be associated with an increased risk of the incidence of CKD (adjusted OR 2.10 (1.16 - 3.76), P < 0.01). Moreover, a one-SD increment in the uric acid level was associated with a 21% increased risk of CKD [10]. Besides the consensus that eGFR could provide a valuable means of stratifying diabetic and non-diabetic patients [12], our present study further validates previous studies in other populations demonstrating the use of uric acid for risk stratification and assessment of renal function [10-12, 23].

Our study differs from previous studies in that, in the present study, the association between serum uric acid and renal function was not consistent with respect to eGFR at baseline and at 6-month follow-up. In patients with eGFR ≥ 60 mL/min/1.73 m^2^, there was no association between serum uric acid level and eGFR at either baseline or at 6-month follow-up. However, in patients with eGFR < 60 mL/min/1.73 m^2^, a significant association was observed between uric acid and eGFR at both baseline and at 6-month follow-up. Moreover, even in the multivariate logistic regression models, in both the third and the highest uric acid quartiles, significant ORs for reduced renal function were observed with or without adjustment for confounding factors. These data reflect to our knowledge what a previously unreported renal function-dependent association between uric acid and eGFR is.

The present study has both strengths and limitations. In terms of strengths, the study cohort contained only subjects in a defined, very-high-risk population diagnosed at a single tertiary hospital in China. Accordingly, our study minimized the impact of potentially confounding variables such as ancestral or genetic background-based differences in CAD or T2DM clinical phenotypes [24]. Second, our study followed a well-designed protocol using well-established methods, with all participants undergoing CAG to ensure accurate diagnosis of CAD. Moreover, only patients with stable CAD and T2DM were enrolled, thereby reducing the influence of unstable disease conditions and complex therapeutic strategies. In terms of limitations, it should be pointed out that the limited study sample size and follow-up period might restrict the extrapolation of our observations to the wider population.

### Conclusions

We have shown for the first time that serum uric acid levels are negatively associated with eGFR under conditions of reduced renal function, and that serum uric acid is an independent risk factor for CKD in individuals with T2DM and stable CAD in the Chinese population. Further studies are required to investigate the impact on renal function and long-term survival in this very-high-risk population of active interventions to decrease levels of uric acid.
